# DOPA: GPU-based protein alignment using database and memory access optimizations

**DOI:** 10.1186/1756-0500-4-261

**Published:** 2011-07-28

**Authors:** Laiq Hasan, Marijn Kentie, Zaid Al-Ars

**Affiliations:** 1 Computer Engineering Laboratory, Faculty of Electrical Engineering Mathematics and Computer Science (EEMCS), Delft University of Technology (TU Delft), Mekelweg 4, 2628 CD, Delft, The Netherlands

## Abstract

**Background:**

*Smith-Waterman (S-W) *algorithm is an optimal sequence alignment method for biological databases, but its computational complexity makes it too slow for practical purposes. Heuristics based approximate methods like FASTA and BLAST provide faster solutions but at the cost of reduced accuracy. Also, the expanding volume and varying lengths of sequences necessitate performance efficient restructuring of these databases. Thus to come up with an accurate and fast solution, it is highly desired to speed up the S-W algorithm.

**Findings:**

This paper presents a high performance protein sequence alignment implementation for *Graphics Processing Units (GPUs)*. The new implementation improves performance by optimizing the database organization and reducing the number of memory accesses to eliminate bandwidth bottlenecks. The implementation is called *Database Optimized Protein Alignment (DOPA) *and it achieves a performance of 21.4 *Giga Cell Updates Per Second (GCUPS)*, which is 1.13 times better than the fastest GPU implementation to date.

**Conclusions:**

In the new GPU-based implementation for protein sequence alignment (DOPA), the database is organized in equal length sequence sets. This equally distributes the workload among all the threads on the GPU's multiprocessors. The result is an improved performance which is better than the fastest available GPU implementation.

## Background

Sequence alignment is used to identify regions of similarity between DNA or protein sequences. This similarity may be a consequence of functional, structural or evolutionary relationships between the sequences. Various methods are available for local and global sequence alignment [1]. Heuristics based approaches like BLAST, FASTA and HMMER [2-4] are fast, but they do not guarantee an optimal alignment. Although slow in aligning long sequences, the *Smith-Waterman (S-W) *algorithm [5], based on *dynamic programming (DP) *[6], is a method that finds an optimal local alignment between two DNA or protein sequences, i.e. the *query sequence *and the *database sequence*. The usual way of aligning sequences is to use algorithms like S-W on a small to medium size clusters of standard CPUs or workstations [7], but the speedup does not increase linearly with the number of CPUs due to issues with workload distribution.

To develop efficient and optimal sequence alignment solutions, the S-W algorithm has recently been implemented on emerging accelerator platforms such as FPGAs, Cell/BEs and GPUs [8-14].

In this paper, we present a high performance GPU-based protein sequence alignment implementation using database and memory access optimizations. The implementation is called DOPA and it pre-converts the reference protein database to a custom GPU format. Like other GPU implementations, the time consuming matrix fill step of the S-W algorithm is implemented and accelerated on the GPU. The performance is enhanced by restructuring the entire database and optimizing its organization.

Furthermore, memory accesses are optimized to eliminate bandwidth bottlenecks. The results demonstrate that DOPA achieves a performance of 21.4 GCUPS, which is 1.13 times better than the fastest available GPU implementation to date.

### GPU as a computational platform

**Figure 1:**
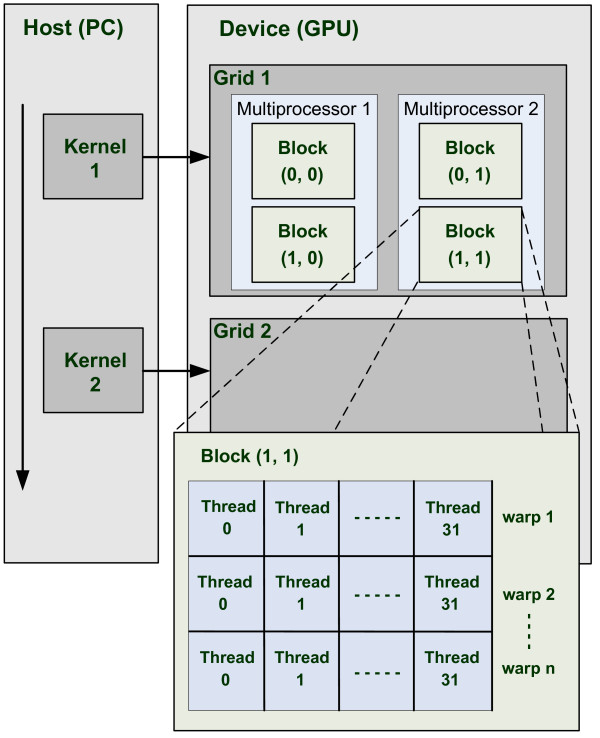
**Programming model**. CUDA hierarchy of threads, blocks and grids.

*Compute Unified Device Architecture (CUDA) *is the hardware and software architecture that enables NVIDIA GPUs [15] to execute programs written in C, C++, Fortran, OpenCL [16], DirectCompute [17], and other languages. A CUDA program calls kernels that run on the GPU, as shown in Figure 1. A kernel executes in parallel across a set of threads, where a thread is the basic unit in the programming model that executes an instance of the kernel, and has access to registers and per thread local memory. The programmer organizes these threads in grids of thread blocks, where a thread block is a set of concurrently executing threads and has a shared memory for communication between the threads. A grid is an array of thread blocks that execute the same kernel, read inputs from and write outputs to global memory, and synchronize between interdependent kernel calls.

CUDA's hierarchy of threads maps to a hierarchy of processors on the GPU. A GPU executes one or more kernel grids. A GPU consists of multiprocessors that execute one or more thread blocks, as shown in Figure 1. Multiple thread blocks can be scheduled by the GPU to run on one multiprocessor sequentially, or in parallel by using thread switching. CUDA cores, i.e. the processing elements within a multiprocessor, execute threads in groups of 32 called *warps*. Performance on GT200-class GPUs can be optimized a great deal by having threads in a half-warp (16 threads) execute the same code path and access memory in a close vicinity.

**Figure 2:**
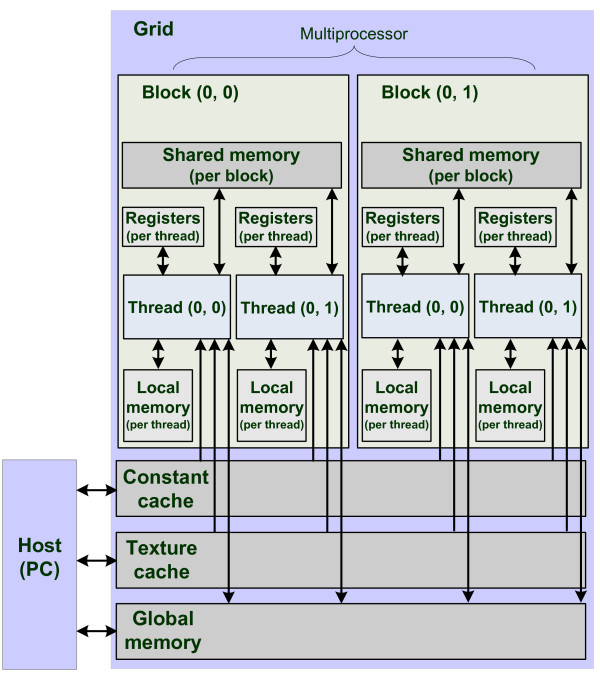
**Memory model**. CUDA memory hierarchy.

In the CUDA parallel programming model various memory spaces exist [15]. The complete set of CUDA memory spaces is given in Figure 2, where global memory is the GPU's RAM. Accessing it has a high latency, which can be hidden by switching execution to other threads that are not waiting for memory accesses.

The second type of memory shown in Figure 2 is the texture cache. Textures are cached 'windows' into global memory, optimized for spatially local reads.

The third type of memory is the constant cache, which is a read-only portion of global memory. It is cached at each multiprocessor and accessing it is as fast as accessing a register.

The other types of memories are shared memory and local memory, where shared memory is a fast memory used for inter-thread communication within a thread block and local memory is a per thread portion of the global memory used for function calls and register spills. Additionally, each multiprocessor offers a bank of registers, shared between its processors.

#### Coalescing

**Figure 3:**
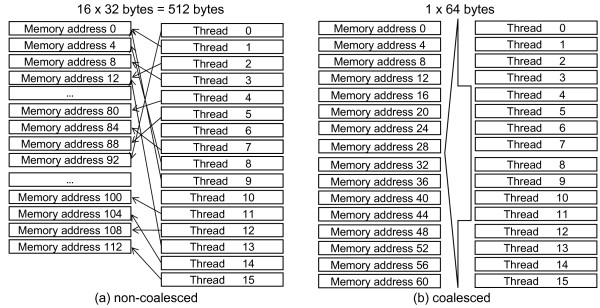
**The effect of coalescing on memory reads**. The figure demonstrates the coalescing phenomenon.

Latency of global memory can be avoided altogether by *coalescing *memory accesses as shown in Figure 3, where each thread of a half-warp of 16 threads accesses a 4-byte value in global memory. The values in Figure 3(a) are all stored at unordered different addresses. In this case, each thread will execute a 32-byte (instead of 4-byte) memory access sequentially, since 32 bytes is the smallest memory access size supported by the GPU. Other possible access sizes are 64 and 128 bytes. This wastes 28 bytes of bandwidth per access adding to a total bandwidth wastage of 28 × 16 = 448 bytes for all 16 threads and as accesses take place sequentially, latency will be high.

In Figure 3(b), the values accessed are stored at neighboring addresses. In this case, coalescing takes place. The GPU issues a single 64-byte load, thus no bandwidth is wasted and only a single access is needed.

### Previous implementations

The first known implementations of S-W based sequence alignment on a GPU are presented in [18] and [19]. These approaches are similar and use the OpenGL graphics API to search protein databases. First the database and query sequences are copied to GPU texture memory. The score matrix is then processed in a systolic array fashion [20], where the data flows in anti-diagonals. The results of each anti-diagonal are again stored in texture memory, which are then used as inputs for the next pass. The implementation in [18] searched 99.8% of Swiss-Prot (almost 180,000 sequences) and managed to obtain a maximum speed of 650 *Mega Cell Updates Per Second (MCUPS) *compared to around 75 for the compared CPU version. The implementation discussed in [19] offers the ability to run in two modes, i.e. one with and one without traceback. The version with no traceback managed to perform at 241 MCUPS, compared to 178 with traceback and 120 for the compared CPU implementation. Both implementations were benchmarked using a Geforce GTX 7800 graphics card.

The first known CUDA implementation, 'SW-CUDA', is discussed in [21]. In this approach, each of the GPU's processors performs a complete alignment instead of them being used to stream through a single alignment. The advantage of this is that no communication between processing elements is required, thereby reducing memory reads and writes. This implementation managed to perform at 1.9 GCUPS on a single Geforce GTX 8800 graphics card when searching Swiss-Prot, compared to around 0.12 GCUPS for the compared CPU implementation. Furthermore, it is shown to scale almost linearly with the amount of GPUs used by simply splitting up the database.

Various improvements have been suggested to the approach presented in [21], as shown in [13,22]. In the 'CUDASW++' solution presented in [13], for sequences of more than 3,072 amino acids an 'inter-task parallelization' method similar to the systolic array and OpenGL approaches is used as this, while slower, requires less memory. This 'CUDASW++' solution manages a maximum speed of about 9.5 GCUPS searching Swiss-Prot on a Geforce GTX 280 graphics card. An improved version, 'CUDASW++ 2.0' has been published recently [14]. Being the fastest Smith-Waterman GPU implementation to date, 'CUDASW++ 2.0' managed 17 GCUPS on a single GTX 280 GPU, outperforming CPU-based BLAST in its benchmarks.

## Methods

The methods used during the development of DOPA, our high performance and optimized GPU implementation for protein sequence alignment, are presented as follows:

### General design

**Figure 4:**
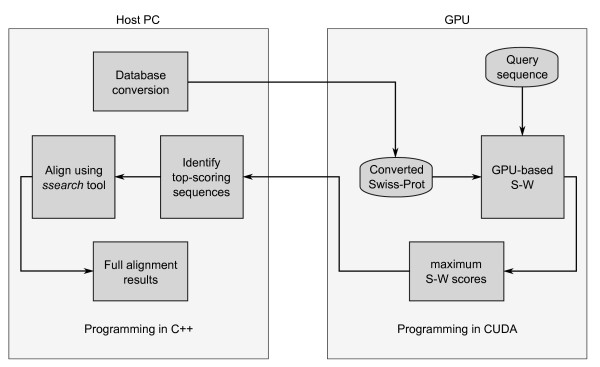
**Description of the DOPA GPU implementation**. The figure presents a block diagram description of the DOPA GPU implementation.

Being the most mature GPU programming toolkit to date, NVIDIA CUDA is used for the GPU programming *(device code) *in conjunction with C++ for the PC programming *(host code)*. Like with other existing GPU implementations, protein sequences from the Swiss-Prot database [23] are considered for alignment. The reason is that protein alignment is more complex than the DNA version, which makes supporting DNA alignments later on relatively simple. In addition, Swiss-Prot has a size of around 300 MB, making it small enough to fit into the global memory of the GPU which is about 1 GB for the device used in this paper. Databases larger than 1 GB have to be divided in chunks before being loaded into the GPU global memory for alignment. Figure 4 shows a block diagram description of the DOPA GPU implementation. The host code is mostly concerned with loading data structures, copying them to the GPU, and copying back and presenting the results. The query sequence, converted database and other data are copied to the GPU. Then the device code is launched, which aligns the query sequence with the database sequences using the S-W algorithm.

Like other GPU implementations, our implementation returns maximum S-W scores instead of the actual alignments. Skipping the algorithm's traceback step significantly simplifies and speeds up the implementation. Furthermore, as no data structures like pointer lists need to be kept, memory consumption is decreased as well. However, to be able to generate full alignments, a number of top-scoring sequences are exported to a new database file. The sequences in this file can then be aligned on the host PC using the *Smith-Waterman search (ssearch) *tool. This approach leads to some redundancy as some sequences are aligned twice, however, the number of such sequences is relatively small. By default 20 top-scoring sequences are returned, whereas the Swiss-Prot database contains more than 500,000. However, the implementation does not limit the number of sequences to be returned. Also, returning more sequences will have negligible effect on performance.

Each processing element in our implementation is used to independently generate a complete alignment between a query sequence and a database sequence. This eliminates the need for inter-processor communication and results in efficient resource utilization. The GPU used for implementation (i.e. NVIDIA GTX 275) contains 240 processors, while the latest release of Swiss-Prot contains more than 500,000 sequences. Hence, it is possible to keep all processors well occupied [24].

### Database conversion

**Figure 5:**
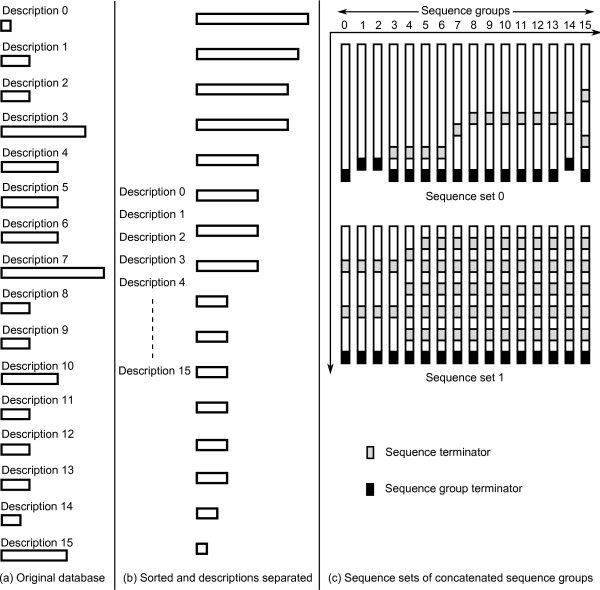
**The database conversion**. The figure illustrates the database conversion process.

In FASTA format, sequences are preceded by sequence descriptions that give names and other biological information about them. Instead of directly loading databases in FASTA format, the GPU implementation converts them to a custom GPU format to better match the device capabilities. A database only needs to be converted once, after which it is locally stored in the new format. The database loading/storing time is around 1.6 seconds. For databases of size larger than the global memory of the GPU, for example 'NR' from NCBI, the converted database needs to be stored locally in chunks. In this case, the loading/storing overhead will be incurred for each stored chunk. The conversion process, as shown in Figure 5, consists of the following steps.

#### Sorting

In practice the threads in a half-warp will have to wait for each other to finish their workload instead of continuing on independently. To reduce this waiting time, the database sequences are sorted by length to minimize length differences between neighboring threads, as shown in Figure 5(b). Sequence descriptions are stored in a separate file that is not uploaded to the GPU, saving memory and decreasing load times. Furthermore, sequence characters are replaced with numeric indexes to facilitate easier substitution matrix lookups.

#### Concatenation

After sorting, groups of 16 sequences are taken and processed in *sequence sets *that will have a half-warp of threads working on them, as shown in Figure 5(c). Even though sorting by length has somewhat equalized workload within each sequence set, various sequence sets still have different sizes. To combat this, sequences within a sequence set are concatenated with leftover sequences to form *sequence groups*. The total length of each sequence group within a sequence set nearly equals or, ideally, matches the length of the longest sequence in that set. This results in an equal workload for each thread in a half-warp processing a sequence set.

*Sequence terminators *are inserted between the concatenated sequences; these tell the GPU kernel to initiate a new alignment. *Sequence group terminators *are inserted at the end of each sequence group signifying the end of a group of concatenated sequences, at which point a thread will wait for the rest of the threads in the half-warp to cease execution.

#### Interlacing

**Figure 6:**
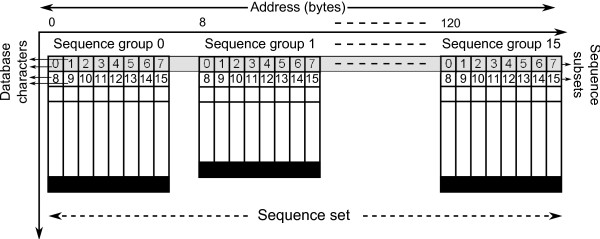
**Interlacing**. The figure shows sequence storing as interlaced subsets.

Once all database sequences have been processed into 16-wide sets of sequence groups, they are written to file. The sequence sets are written in an interlaced fashion, as shown in Figure 6. Each interlaced *subset *consists of eight characters from each sequence group.

Eight characters of the set's first sequence group are written, then eight characters of the set's second group and so on. As there are 16 sequence groups in each sequence set, each thread in a half-warp is now able to load 8 bytes of sequence data from neighboring addresses. As a result, 128-byte coalesced loading takes place.

#### Equal length sets

During code development, alignments were conducted with a synthetic (randomly generated) database, each sequence of which had the same length. The performance of this synthetic database is twice that of the Swiss-Prot database, which has sequences ranging in length from 2 to 35213 characters. The drop in performance for Swiss-Prot is the result of different workloads between different half-warps.

Though concatenation resulted in an equal workload distribution for threads within every sequence set, it still varies among different sequence sets. To resolve this, the length of each sequence group within every sequence set is made equal or nearly equal to the length of the longest sequence in the database, as shown in Figure 5(c). This results in an equal workload distribution for all GPU threads in general. The outcome of this is a 1.7 times increase in performance.

Evidently, equal workload across different threads improves performance; possibly a result of the GPU's thread scheduling not being optimal in the previous case. For example, the GPU thread scheduler might only schedule a new thread block once all the threads in a previous thread block have completed their execution.

### Temporary data reads and writes

Memory bandwidth represented a serious bottleneck while developing the GPU implementation. A number of steps have been taken to optimize for high performance by reducing the number of memory accesses, the frequent temporary data accesses in particular.

The S-W algorithm with affine gap penalties [25] is given by Equation 1, where *α*, *β *are the gap opening and extension penalties, respectively. In contrast to the regular or linear gap penalties, an affine gap penalty encourages the extension of gaps rather than the introduction of new gaps. Further, *H*_0,0 _= *D*_0,0 _= *E*_0,0 _= *H*_*i*,0 _= *D*_*i*,0 _= *E*_*i*,0 _= *H*_0,*j *_= *D*_0,*j *_= *E*_0,*j *_= 0, for 1 ≤ *i *≤ *M *and 1 ≤ *j *≤ *N*, where *M *and *N *are the lengths of the sequences to be aligned.(1)

As no traceback is performed on the GPU, S-W matrix values do not need to be saved for the entire execution time and can be overwritten. As such, only a single column of S-W scores is kept. This score column stores values to the left of the currently processing column, i.e. *H*_*i -*1, 1 ≤ *j *≤ *N *_in Equation 1. The size of this temporary data column is set to the size of the query sequence, not the database sequence, so that the column can have one fixed size for all database sequences. This usually requires less memory, as it is unlikely that the query sequence will be as long as the longest database sequence. The temporary data column is set to zero whenever a new database sequence is started. In addition to this temporary score column, variables are used to keep the values of the upper and upper-left cells required by the algorithm, i.e. *H*_*i*,*j *- 1 _and *H*_*i - *1,*j - *1 _in Equation 1. To support affine gap penalties, another temporary data column is added for *D *values. Additionally, an upper *E *value is kept (see Equation 1).

Each S-W iteration involves reading and writing two temporary values (score and *D*), for four accesses in total. When both are non-coalesced, 32 byte reads/writes are issued for each access. This means that per half-warp

of bandwidth is used, resulting in a major memory bottleneck. The optimization steps mentioned below decrease this to one 128-byte coalesced read and write for every second iteration. This is a 16 times bandwidth improvement and requires only 1 instead of 64 accesses. 128 bytes is the largest allowed coalesced access size, and is faster than multiple smaller coalesced accesses [24]. The optimizations are as follows:

• Smaller, 16-bit data type for the temporary values, cutting the theoretically required bandwidth in half and allowing for better coalescing.

• Each thread stores one data value in turn, resulting in an interlaced storage scheme. Instead of direct array accesses, a pointer into the temporary storage is started at the thread *id*, and increased by the total number of threads to move to the next element of the *H *matrix. Each thread in a half-warp then reads a 2-byte coalesced value, meaning that instead of two 32-byte accesses per thread, two such accesses take place per half-warp. This sixteen times bandwidth improvement results in an almost ten times net speedup.

• To again halve the number of memory accesses, the temporary score and *D *values are interlaced. This is done by defining a data structure consisting of these values and using it to access the score and *D *values for an iteration in one go. At this point, a thread accesses two 2-byte values in one read, for a total of 16 × 2 × 2 bytes bandwidth per half warp. The result is a 64-byte coalesced access.

• Finally, two temporary values are interlaced to move to 128-byte accesses. This has an additional benefit of temporary reads/writes only being required for every second query sequence symbol processed.

### Substitution matrix accesses

Aligning proteins requires the use of a substitution matrix, which is accessed every time two symbols are aligned, making its access time critical to the implementation's performance. Substitution matrix (e.g. BLOSUM 62) accesses are random and are completely dependent on the database sequence, complicating the choice of memory used. Global memory is not a good choice for such a frequent usage due to its high access time. Also the random nature of substitution matrix accesses makes coalescing very difficult. As an alternative, the substitution matrix is stored in texture memory. Texture memory is a cached window into global memory that offers lower latency and does not require coalescing for best performance. It is thus well suited for random access. Texture memory has the ability to fetch four values at a time. This mechanism can be used to fetch four substitution matrix values from a *query profile*.

**Figure 7:**
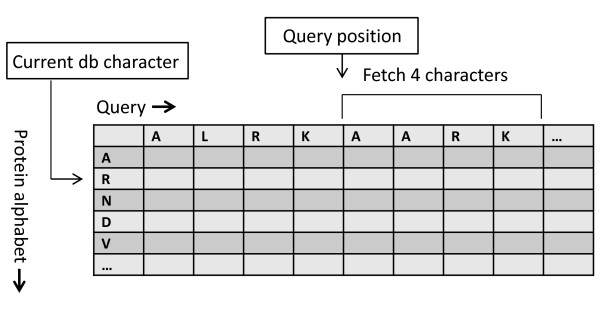
**Query profile**. The figure describes memory accesses to fetch values from a query profile.

A query profile is shown in Figure 7. It is a type of substitution matrix where, instead of the protein alphabet, the query sequence is used along the top row. This means that for a given database character, the substitution matrix is not random anymore: multiple substitution scores can be loaded simultaneously when aligning the query with a database character. Furthermore, query sequence lookups are not required anymore; only the current position within the query is needed to index into the profile. A query profile is generated once for every query sequence. Each query profile column stores values for 23 characters. The number of columns and hence the memory requirement for a query profile depends on the length of the query sequence. The GTX 275 GPU used for our implementation has 8KB of texture cache per multiprocessor. This means that a query sequence having more than ⌊8 × 1024/23⌋ = 356 characters will result in increased cache misses, as described in [22]. Tests were performed to quantify the texture cache miss rate, which was shown to be very small. For example, aligning an 8000 character query sequence resulted in 0.009% miss rate. Using this query profile method resulted in a 17% performance improvement with Swiss-Prot [24].

## Results and Discussion

Following is a discussion of results which presents the experimental setup used for development, testing and measuring the performance of DOPA. The performance of DOPA is evaluated and compared with other available approaches.

### Experimental setup

The experimental setup used to test the implementation and measure its performance is as follows:

• Intel Core 2 Quad Q6600 (2.4 GHz) with 4 GB of RAM

• NVIDIA Geforce GTX 275 graphics card with 896 MB of memory and clock speeds of 633, 1134 and 1404 MHz for its core, memory and shaders respectively

• 64 bit Microsoft Windows 7 Professional

• Video drivers version 257.21

• CUDA toolkit version 3.1

• Swiss-Prot release October 2010

• Substitution matrix BLOSUM62

• Gap penalty: -10 and gap extend penalty: -2 (these do not influence the execution time)

The run time is measured using the C clock() instruction, the accuracy of which is verified using the CUDA profiling application. Table 1 displays the performance results, where the execution time in seconds and the performance in GCUPS are given for query sequences of varying lengths taken from Swiss-Prot and aligned against the same database.

**Table 1:** Performance results with Swiss-Prot

Query sequence	Length	Execution time (seconds)	Performance (GCUPS)
P02232	144	1.24	21.35

P05013	189	1.65	21.06

P14942	222	1.93	21.15

P07327	375	3.24	21.28

P01008	464	3.99	21.38

P03435	567	4.89	21.32

P27895	1000	8.60	21.38

P07756	1500	12.91	21.36

P04775	2005	17.27	21.35

P19096	2504	21.54	21.37

P28167	3005	25.88	21.35

P0C6B8	3564	30.67	21.37

P20930	4061	34.97	21.35

Q9UKN1	5478	47.15	21.36

The table displays performance results, where execution time in seconds and performance in GCUPS are given for query sequences of varying lengths taken from Swiss-Prot and aligned against the sequences in the same database.

**Figure 8:**
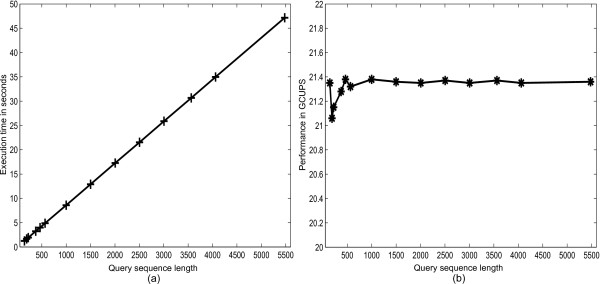
**Performance results with Swiss-Prot**. The figure shows execution time and performance for query sequences of varying lengths.

Figure 8(a) shows that the execution time increases linearly with sequence length, resulting in an almost constant performance of around 21.4 GCUPS, shown in Figure 8(b).

### Performance comparison

**Figure 9:**
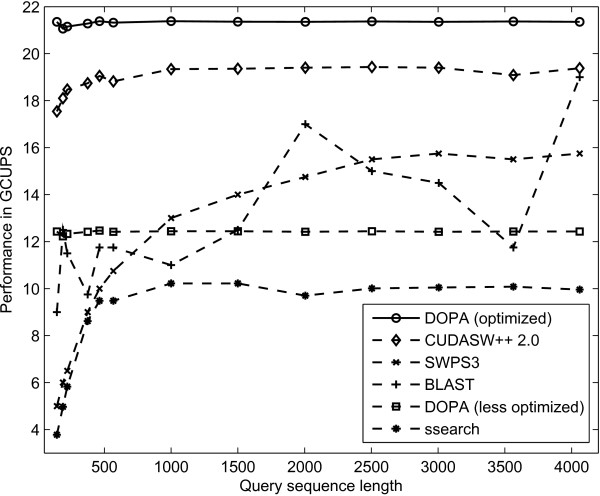
**Performance comparison**. The figure shows a comparison of the fully optimized version of our implementation (optimized DOPA) with CUDASW++ 2.0 [14], i.e. the fastest GPU-based Smith-Waterman implementation to date, a vectorized S-W implementation on PlayStation3 (SWPS3), BLAST, a less optimized version of our implementation with no equal length sequence sets (less optimized DOPA) and an accelerated and multi-threaded version of *ssearch (SSE2)*.

The optimized version of our implementation is compared with: a multi-threaded high performance *ssearch (SSE2)*; a less optimized version of our implementation with no equal length sequence sets; and with CUDASW++ 2.0 [14], the fastest GPU-based Smith-Waterman implementation to date. The comparison is shown in Figure 9 and described as follows.

#### Comparison with ssearch

*Ssearch (SSE2) *is an accelerated and multi-threaded version of ssearch, where ssearch is a CPU-based Smith-Waterman alignment tool that can be found in the FASTA suite of applications [26]. The SSE2 optimizations, described in [27] utilize modern CPU's vector extensions for a performance increase. The ssearch is run on the same system, using the same experimental setup as DOPA. The results demonstrate that our implementation performs 2.14 times better in terms of GCUPS than this accelerated and multi-threaded version of ssearch.

#### Comparison with a less optimized version

In the less optimized version, only some of the database optimization steps mentioned in the Methods section have been performed. In this version, sequences are only sorted, concatenated and interlaced. However, no equal length sets were used, making the length of each sequence set depend on the longest sequence within that set. When run on the same experimental setup, this less optimized version results in a performance of around 12.5 GCUPS. The comparison shows that our fully optimized GPU implementation performs around 1.7 times better than the less optimized version. This demonstrates the performance impact of the crucial optimization of equal length sequence sets, which results in an improved scheduling.

#### Comparison with BLAST

DOPA is also compared with a vectorized version of the heuristic method (NCBI - BLAST) [28] running on a 2.4 GHz Intel Core 2 Quad processor. The results show that DOPA performs 1.8 times better in terms of GCUPS than vectorized BLAST on the average, as shown in Figure 9.

#### Comparison with SWPS3

*Smith-Waterman on a PlayStation 3 (SWPS3) *is an optimized and vectorized CPU implementation of the Smith-Waterman that calculates the alignment scores [28] and is faster than the SSE2 implementation of FASTA ssearch. While running under the same experimental setup, DOPA performs around 1.43 times better in terms of GCUPS than the optimized vectorized SWPS3, as shown in Figure 9.

#### Comparison with CUDASW++ 2.0

CUDASW++ 2.0 is the fastest GPU implementation to date for S-W based protein sequence alignment. CUDASW++ 2.0 runs in two stages, i.e. one for short sequences and the other for long sequences. The first stage for aligning short sequences works by using an approach similar to DOPA but without using concatenation and equal length sequence sets. The second stage used for aligning long sequences works by using a systolic array based alignment approach. Long sequences in a database inherently have the largest length differences, which is specifically true for Swiss-Prot. Thus, aligning them using the systolic array based approach reduces the workload differences. The sequence length beyond which CUDASW++ 2.0 switches from the first to the second stage is defined by the user.

When run on the same system using same experimental setup as DOPA, CUDASW++ 2.0 achieves a performance of around 19 GCUPS. Thus our fully optimized implementation performs 1.13 times better than CUDASW++ 2.0 in terms of GCUPS. Both approaches are sensitive to the structure of the database used. Like our implementation, CUDASW++ 2.0 also uses 16-bit score values, as discussed in the Methods section.

Table 2 summarizes the optimization steps undertaken by our fully optimized and less optimized DOPA implementations in comparison with CUDASW++ 2.0. In comparison with CUDASW++ 2.0, our less optimized implementation performs 1.52 times slower in terms of GCUPS, as shown in Figure 9. This is specifically due to their two stage implementation. In the fully optimized version, we overcome this deficiency by introducing equal length sequence sets resulting in an equal workload distribution and improved performance.

**Table 2:** A comparison with CUDASW++ 2.0

**No**.	Optimizations	CUDASW++ 2.0	DOPA (less optimized)	DOPA (optimized)
1	Database sorting	+	+	+

2	Concatenation into sequence groups	-	+	+

3	Interlacing	+	+	+

4	Equal length sequence sets	-	-	+

5	Query profile	+	+	+

The table compares the optimization steps adapted by DOPA and CUDASW++ 2.0.

Additionally, DOPA also brings in the following improvements:

• In comparison with CUDASW++ 2.0, DOPA is simpler, as it uses just one search kernel instead of two, requiring no inter-processor communication.

• The optimized database organization scheme used in DOPA allows an equal workload for each thread block, while CUDASW++ 2.0 uses a hand-picked point at which it switches from one kernel to the other for its work distribution.

• DOPA is complete and usable, not just a proof of concept, as it exports the top-scoring sequences for full alignment with *ssearch*. CUDASW++ 2.0 does not provide this facility. Our implementation also provides a web interface that allows it to be used conveniently and remotely.

## Conclusion

This paper presented a high performance GPU-based implementation for protein sequence alignment. The new implementation, called DOPA, improves the performance by reducing the number of memory accesses and optimizing the database organization. The database is organized in equal length sequence sets resulting in an equal workload distribution for all the threads of each multiprocessor on the GPU. The performance achieved by DOPA is 21.4 GCUPs. When compared with the fastest available GPU implementation for protein sequence alignment to date, DOPA reports a 1.13 times improvement in terms of GCUPS.

## Availability and Requirements

**Project name: **DOPA

**Project home page: **http://kentie.net/article/thesis/index.htm

**Operating system: **64 bit Microsoft Windows 7 Professional

**Programming language: **C++ and CUDA

**Other requirements: **CUDA toolkit version 3.1 or higher

## Competing interests

The authors declare that they have no competing interests.

## Download Source

All the necessary files are available for download at http://kentie.net/article/thesis/index.htm

## Authors' contributions

LH carried out the study, participated in the algorithm optimization and analysis of the results and drafted the manuscript; MK carried out the design and implementation of the algorithm, performed benchmark tests and participated in the algorithm optimization and analysis of the results; ZA proposed and supervised the study and contributed to the revising of the manuscript. All authors read and approved the final manuscript.
